# Draft Genome Sequence of the *Bordetella bronchiseptica* Swine Isolate KM22

**DOI:** 10.1128/genomeA.00670-14

**Published:** 2014-07-10

**Authors:** Tracy L. Nicholson, Sarah M. Shore, Darrell O. Bayles, Karen B. Register, Robert A. Kingsley

**Affiliations:** a National Animal Disease Center, Agricultural Research Service, Department of Agriculture, Ames, Iowa, USA; b Institute of Food Research, Norwich Research Park, Norwich, United Kingdom; c The Wellcome Trust Sanger Institute, Hinxton, Cambridge, United Kingdom

## Abstract

*Bordetella bronchiseptica* swine isolate KM22 has been used in experimental infections of swine as a model of clinical *B. bronchiseptica* infections within swine herds and to study host-to-host transmission. Here we report the draft genome sequence of KM22.

## GENOME ANNOUNCEMENT

*Bordetella bronchiseptica* colonization is widespread in swine populations and is an important contributor to respiratory disease in pigs. In young pigs, it is a primary cause of bronchopneumonia and in older pigs contributes to secondary pneumonia. It is the primary etiologic agent of nonprogressive atrophic rhinitis, a mild to moderately severe reversible condition, and it promotes colonization by toxigenic strains of *Pasteurella multocida*, which leads to severe progressive atrophic rhinitis (1–3). In pigs exhibiting pneumonia, *B. bronchiseptica* is often isolated in combination with other pathogens (3, 4). It is well documented that co-infection with *B. bronchiseptica* increases colonization and exacerbates the severity of disease caused by both viral and bacterial pathogens, including swine influenza virus, porcine reproductive and respiratory syndrome virus, porcine respiratory coronavirus, *Haemophilus parasuis, P. multocida*, and *Streptococcus suis* (3, 5–12).

The virulent *B. bronchiseptica* swine isolate KM22 was originally isolated in Hungary in 1993 from a swine herd with atrophic rhinitis. Based on multilocus sequence type (MLST) analysis, KM22 is sequence type (ST) 7, in Clonal Complex 1 of an MLST-based *Bordetella* phylogeny (13) and harbors a ribotype (14) and pertactin repeat region variant (15) shared with the majority of strains isolated from swine. KM22 has been successfully used by our laboratory to develop a reproducible swine respiratory disease model reflective of clinical *B. bronchiseptica* infections within swine herds and host-to-host transmission (2, 5–10, 16–19). Here we report the draft genome sequence of KM22 in our continued efforts to investigate the pathogenesis and transmission of *B. bronchiseptica* in swine.

DNA for whole genome sequencing was prepared from bacteria grown overnight at 37°C in Stainer-Scholte (SS) broth and genomic DNA was prepared using a High Pure PCR template preparation kit (Roche Applied Science, Indianapolis, IN) according to manufacturer’s instructions. Sequencing was performed using a combination of Roche GS (FLX and FLXplus) and Illumina GAIIx sequencing. The use of two Roche GS (FLX and FLXplus) genomic shotgun libraries and sequencing resulted in the generation of 891,959 reads with an average length of 248 bp (for FLX) and 1,209 bp (for FLXplus). Illumina GAIIx sequencing resulted in a total of 3,474,442 paired-end sequencing reads of 72-bp length from a template of 239 bp average insert size. The genome was assembled using MIRA v3.4.0 and the Roche GS *De Novo* Assembler v2.6 to achieve 87× total genome coverage through the assembly of Roche GS FLX shotgun, GS FLXplus shotgun, and Illumina GAIIx paired-end sequencing reads. *B. bronchiseptica* RB50 was subsequently used as a reference to further guide closing genome gaps via manual editing. Gap5 from the Staden package was used as the assembly editor resulting in a final assembly of 46 contigs consisting of a maximum length of 679,114 bp, a minimum length of 686 bp, an *N*_50_ contig size of 277,171 bp, a mean coverage of 87×, and summing to a total genome size of 5,119,729 bp. Automated annotation using Prokka v1.7 (20) identified a total of 4,853 predicted protein coding sequences (CDSs), 3 rRNA operons, 1 transfer-messenger RNA (tmRNA), and 54 tRNAs.

### Nucleotide sequence accession numbers.

The Illunina HiSeq short read sequence has been deposited at the European Nucleotide Archive with the accession no. ERS027415. This whole-genome shotgun project has been deposited in DDBJ/ENA/GenBank under the accession no. JNHR00000000. The version described in this paper is the first version, JNHR01000000.
